# Nasogastric tube feeding under physical restraint on paediatric wards: ethical, legal and practical considerations regarding this lifesaving intervention

**DOI:** 10.1192/bjb.2022.11

**Published:** 2023-04

**Authors:** Sarah J. Fuller, Simon Chapman, Emma Cave, James Druce-Perkins, Poppy Daniels, Jacinta Tan

**Affiliations:** 1 Advanced Specialist Eating Disorders Dietitian, East London NHS Foundation Trust and Child; and Child and Adolescent Mental Health Department, Imperial College London, UK; 2 Consultant in Paediatric and Adolescent Medicine, Department of Paediatrics, King's College London, UK; 3 Professor of Healthcare Law, Department of Paediatrics, Durham University, UK; 4 Highly Specialist Dietitian, Child and Adolescent Mental Health Department, Aneurin Bevan University Health Board; 5 expert by experience; 6 Consultant Child and Adolescent Psychiatrist, Oxford Health NHS Foundation Trust; and Senior Clinical Research Fellow, Department of Psychiatry, Oxford University, UK

**Keywords:** Nasogastric tube feeding, restraint, legal, ethics, paediatrics

## Abstract

Eating disorders have the highest mortality rate of any psychiatric condition. Since the COVID-19 pandemic, the number of patients who have required medical stabilisation on paediatric wards has increased significantly. Likewise, the number of patients who have required medical stabilisation against their will as a lifesaving intervention has increased. This paper highlights a fictional case study aiming to explore the legal, ethical and practical considerations a trainee should be aware of. By the end of this article, readers will be more aware of this complex issue and how it might be managed, as well as the impact it can have on the patient, their family and ward staff.

## Clinical scenario

You are a trainee with the local Child and Adolescent Mental Health Service (CAMHS) team and are joining the consultant psychiatrist reviewing a 14-year-old female patient who is currently on a local paediatric ward in England. The patient was referred to her local CAMHS team 9 months ago, presenting with self-harm and anxiety. Initially, she engaged well with CAMHS and was working to reduce her self-harm.

Three months ago, her CAMHS team noted that she was losing weight and voicing anorexic cognitions (such as wanting to be thin and thinking she was fat), and she subsequently was assessed by the eating disorders CAMHS team 2 months ago and diagnosed with atypical anorexia nervosa, as her weight on assessment was above the normal range (115% median body mass index (BMI)). Family-based treatment was started but, over the past 8 weeks, she has continued to lose weight as her parents have found it difficult to engage in this treatment because they view her weight loss positively and do not think her eating is a problem at present. However, in the past week, the patient's family have reported that she is no longer eating at home and has lost more weight, fainted twice and is only drinking a cup of water a day. The patient has lost 8 kg in the past 5 weeks and is now at 92% median BMI (approximately 25th centile). She is saying that she would rather die than eat. Her parents, having previously felt that an admission was not needed, now feel that it is, and she was admitted 3 days ago. She has continued to refuse to eat or drink, pulled out her cannula for intravenous fluids this morning and is refusing nasogastric tube (NGT) feeding.

There are concerns around her deteriorating physical health: her pulse is 39 beats per minute in the day and 29 beats per minute at night, her temperature is 34.7°C, her electrocardiogram shows an elongated corrected QT interval at 460 ms and her biochemistry is showing that her kidney function is compromised owing to dehydration. The eating disorders team have made a referral to the local adolescent mental health unit; however, there is a waiting list for an admission and there are no other beds available nationally. Furthermore, the nurses at the adolescent mental health unit say they cannot nurse anyone who is not medically stable.

## Summary box

Questions to consider:

- How can we safely refeed and rehydrate a young person who is refusing nutrition and fluids?
- What are the legal and ethical considerations this case brings?
- What support is needed to safely manage this patient's admission to the paediatric ward?

## Discussion

In this case, the young person has lost a significant amount of weight (23%) in a short period of time, while being seen by CAMHS and the eating disorders team, despite her current weight being within a range of normal for many young people. Her physical health is significantly compromised and there is a risk that she may deteriorate further or die if her starved and dehydrated state were to continue. The paediatric team would like to discuss with yourself and the eating disorders team if it would be appropriate to use NGT feeding under restraint, to refeed and rehydrate her as a lifesaving intervention.

NGT feeding is when a tube is placed through the patient's nose and oesophagus, and allows for fluids and nutrition to be passed directly into their stomach. NGT feeding is usually used in medical and paediatric wards for patients who cannot safely swallow, are unconscious or have nutritional requirements that cannot be met with oral diet alone. However, when a patient is refusing to eat or drink as part of a mental health condition, and their life is at risk, the appropriate legal frameworks can be used to facilitate this under physical and/or pharmacological restraint if it has not been possible for the patient to accept this intervention.

## Current literature

To date, there has been little research regarding NGT feeding under restraint in mental health patients, and there is a significant gap in the research regarding nursing, pharmacological or psychiatric approaches to NGT feeding under physical restraint. Papers have highlighted the patient groups that may require this intervention,^1,2^ and there are dietetic guidelines regarding how practice can be modified in line with the legal principle of least restrictive practice.^3,4^ One paper highlights the potential traumatic risks of compulsory treatment, and although it does not specifically focus on NGT feeding under physical restraint, it does reflect that these patients, once fully recovered, are often grateful for compulsory treatment and all that it entailed.^5^ One qualitative paper has focused on trying to understand the meaning of NGT feeding in patients with anorexia nervosa, although again this was not specifically in relation to NGT feeding under physical restraint, and this revealed that it could mean different things for different patients.^6^ Broadly, NGT feeding could be characterised as an unpleasant experience, a necessary and helpful intervention, a signifier of anorexia nervosa or a reflection of the struggle of control during treatment.^6^ Research also suggests that NGT feeding as an intervention for patients with anorexia nervosa carries the risk of self-sabotage and non-adherence in up to 30% of patients.^7^

## Considerations and reflections

### Legal considerations

The law seeks to act in the best interests of the young person and uphold their human rights. If persuasion to drink and eat fails, and it is not otherwise possible to treat her safely, her deteriorating physical health will lead to the consideration of compulsory treatment. Sometimes the suggestion that treatment might be lawfully compelled will be enough to secure adherence, in which case a child who is Gillick competent^8^ can consent to informal admission and treatment, or treatment at home. Gillick competence is assessed in relation to her maturity and understanding and in respect of the decision. If the young person lacks Gillick competence and is adherent by the time treatment is urgently needed, those with parental responsibility (usually the parent/s) may consent on her behalf.

If, however, the young person remains resistant to urgently needed treatment, then in England and Wales parents have limited powers to consent against her wishes. If the person makes a Gillick-competent refusal, their parents cannot provide the requisite consent and veto their decision.^9,10^ Even if they lack competence and their parent/s are willing to consent to admission and treatment, guidance restricts parents to making decisions within the ‘scope of parental responsibility’, although the matter has not been fully tested in the courts.^11^ The Mental Health Act (MHA) Code of Practice states that parents will not be able to make decisions in relation to the medical care of the child if they are not the kind of decisions parents routinely make. This is dependent on many factors, including age, stage of development and the circumstances including the level of invasiveness. Consent to invasive treatment, such as NGT feeding under restraint, would almost certainly fall outside the scope of parental responsibility.^10^

If the young person's health is at serious risk, and they withhold consent yet treatment in the paediatric ward is the only safe option, then compulsory detention for assessment (Section 2) or treatment (Section 3) under the MHA 1983 may be appropriate.^12^ The grounds set out in Section 2 or 3 of the MHA 1983 must be met. Under Section 2, the person must be potentially suffering from a mental disorder of a nature or degree that warrants their detention in hospital, and detention must be in the interests of the person's own health, their safety or for the protection of other people. Treatment can be delivered compulsorily under Section 2, as part of the assessment. Under Section 3, it must be necessary for the health or safety of the young person that they receive treatment that cannot be provided if the patient is not detained. In all cases, the young person's wishes and views, social and family circumstances should be considered, as should the impact on the young person of detaining them under the MHA for treatment.

## Ethical considerations

From an ethical point of view, there are many different considerations that need to be balanced in every decision to feed under restraint. Many young people who are highly distressed may also be impaired in their competence to make treatment decisions, as are some patients with severe eating disorders and malnutrition. In eating disorders, competence is often specific to treatment decisions, which will potentially cause weight gain, with patients otherwise perfectly capable of making other decisions. A calm, considered and compassionate approach can increase the chance of a peaceful resolution, as can patients having trusting relationships with clinicians and families.^13^

Every patient has a right to be heard and express their wishes, irrespective of whether their decisions are made competently.^10^ Regardless of competence, the MHA can be used in England and Wales to override treatment refusal. At the same time, care needs to be taken to respect autonomy as much as possible and inform the patient of what is planned, and care is needed to use the least restrictive measures possible and minimise repeated use.

Patient dignity and respect must be prioritised, especially in carrying out such intrusive and uncomfortable procedures. This can take the form of giving the patient adequate warning of each impending feed, and maximal choice and support to decide to eat or drink to avoid feeding under restraint altogether; ensuring it happens in a private place away from other patients; careful explanation of each step of the process before it is done; using minimal force or restraint, and provision of sedation if this is helpful to reduce distress or the patient wishes it.

There must always be enough trained staff to carry out the process safely, competently and kindly, and they should work well together to deliver it. NGT feeding under restraint must never be punitive or routine; it should always be the last resort in a situation of necessity, done calmly and efficiently to minimise restraint. Although this intervention is a highly distressing event for all, it can be necessary to save life, and protecting life is a paramount consideration enshrined in the European Convention of Human Rights as the right to life, which is particularly important in legal minors for whom welfare is paramount.^10,14–16^

Parents can be distressed and may feel helpless or angry in situations where there is refusal of food and drink and coercive feeding is needed, and experience a mixture of fear for their child's life and horror at the use of coercive feeding. Use of the MHA and delivery of the feeding by competent clinicians can be a relief to parents, and indeed patients themselves, if the message that is conveyed is ‘we care, and we will not let a young person die’, which can relieve the young person and parents of the responsibility for the decision to eat or not eat. Patients and parents do appreciate compulsory treatment, despite objections, when they understand that treatment is being done to save life and is in their best interests.^5^ The intrusiveness of the process means that it can be unfair for parents to be asked to make decisions that may earn them the lasting resentment of their child or damage relationships. If coercion is needed, it is more appropriate that this is done dispassionately by clinicians under direction of a psychiatrist or paediatrician who can assume the role of the ‘bad guy’, by taking responsibility for the compulsion in this emotionally fraught scenario. For restrictive and coercive measures such as NGT feeding under restraint, honest acknowledgement should be made of the traumatic and distressing experience it can be for patients, parents and staff. All three groups may need support and the opportunity to debrief and address their feelings both at the time and after.

## Expert by experience reflection

Informed and written consent has been obtained from our expert by experience: ‘Although I don't remember the event clearly, I was NGT fed under resistant as a 17-year-old on an eating disorders unit. It was horrible to have part of my free will stripped away from me, but looking back, there were no alternatives. I was too unwell to think logically, I'd completely given up and didn't understand or care what the consequences were. Eight years on, I'm grateful. I'm in my second year of studying medicine and have been fully recovered from anorexia for years.

It terrifies me to think of how bad the situation now is that children are being NGT fed under resistant on paediatric wards, which must make an already traumatic experience even worse.’

## Clinician reflection from a specialist eating disorders dietitian

‘Previously, we had never needed to NGT feed a patient under physical restraint on our paediatric wards, thinking it was something that only happened in mental health in-patient units. Sadly, during the COVID-19 pandemic, we have experienced this with an increased number of patients.

As a team, we felt a shared sense of relief that our patient was finally receiving the appropriate, lifesaving nutrition and fluids that they needed. This gave us hope. Hope that it would help the patient break out of the starved state and be able to engage in treatment. At the back of everyone's mind is the concern that our patient may die.

The decision-making process, however, would often take time and cause anxiety among our team, the paediatric team, the patient and their family. There are risk frameworks to help identify who may need admission to the wards, but this does not state at what point a patient's life is at such risk that we need to consider this lifesaving intervention.^17^ Different clinicians have different thresholds of when to act. Once the decision was made, having the patient assessed under the MHA takes time, leading to further anxiety around the patient's deteriorating medical state. Then, the logistics become an issue, resulting in frantic calls to agency staff who are trained in physical restraint, to ensure the intervention was carried out safely.

Physically restraining children and young people is not common practice on paediatric wards, and the staff often report how difficult the experience was for them’.

## Practical considerations

### Questions to consider

Important questions to consider asking when assessing if a patient should be NGT fed against their will:

- What are the indications that this patient is so medically unwell that this intervention should be considered? Is there organ compromise? Is the patient's nutrition, hydration and biochemical state deteriorating?
- Is there the possibility that we (the teams) can wait a few days to see if the patient starts to eat and drink even if this is suboptimal?
- Have the medical issues and severity of the situation been clearly discussed with the young person and their family in a way that they fully understand the seriousness of the situation?
- Can we negotiate an oral intake that is sufficient to prevent further deterioration in their physical state?
- Would detention under the MHA allow this patient to consent to NGT feeding?
- Should the NGT stay in place between feeds?
- What should happen if there is life-threating deterioration in the patient's physical state while waiting for the MHA assessment?

### Adapting dietetic practice

If the above questions and extensive multidisciplinary discussions indicate that this intervention is needed, there are some adaptations to standard paediatric practice that you should be aware of. We do not advise the use of enteral pumps, which would normally be used with NGT feeding, because they require prolonged restraint that is likely to increase distress and conflict. Dietetic practice can be adapted^3^^,^^4^ to ensure that delivery of the required nutrition can happen safely over a short period, to minimise the distress the patient experiences from being fed this way.

Joint working with the paediatric and dietetic team is essential to prescribe the appropriate nutrition and fluids, as this patient will also be at risk of refeeding syndrome. Refeeding syndrome describes a potentially fatal shift in electrolytes and fluids in malnourished patients when nutrition is restarted.^18^ The patient should become medically stable within a few days with consistent and adequate nutrition and hydration, and it would be appropriate to stop NGT feeding under physical restraint when this is achieved.

The NGT should stay in place between feeds unless there is a risk of ligature or if there are attempts to tamper with the tube. Removing the NGT after each feed would not be in line with MHA principle of least restrictive practice. In some cases, it may be appropriate to remove the NGT between feeds, especially if NGT feeding is only being used occasionally, and this may act as a motivation to fully establish a sufficient oral diet.

### Getting the right balance between reflection and action

Even when the situation is urgent, it is worth attempting to de-escalate conflict, reduce expressed (negative) emotion and avoid use of NGT feeding under restraint. This should involve spending time with the patient, building relationships and understanding their motivations and reasons for refusal of food and fluids, as this would allow clinicians to engage the patient in negotiations. It is especially important to involve their parents by explaining the situation, addressing their concerns and hearing their views, both during the crisis and on an ongoing basis. Furthermore, it is key to enable reflection among clinicians of the dynamics and factors involved including determination of best interests. It is important to achieve consensus within both the paediatric team and CAMHS/eating disorders team consult, as the decision-making process needs to be joint across both teams. Where possible, decisions should be delayed to see if the conflict can be resolved, and experts can be consulted as needed, before NGT feeding under restraint is embarked upon. There needs to be a balance between reflection and action: it is rare that NGT feeding under restraint cannot wait an hour, but a decision about a young person who is not eating or drinking may not be able to wait an entire weekend to resolve. Likewise, if NGT feeding under restraint is required and medical stabilisation achieved, it would be appropriate to stop this intervention and ‘wait and see’ what happens. Will the young person accept feeds orally, or start to eat or drink supplements instead? If the young person continues to refuse to eat and drink again and becomes medically unstable to the same extent, NGT feeding under restraint may need to be reinstated.

### Interpreting the clinical signs

When working alongside paediatricians it is important to identify whether they have a nominated lead for patients with eating disorder. All paediatricians will be confident to identify the acute medical risks of dehydration; not all are as confident assessing the risks related to starvation^17^ or the nuanced interpretation of normal blood, examination and physical measurement results within this patient group. For example, a normal set of electrolytes can distract the clinician from the frail physical state. More subtle findings in the results that can signpost weight loss may be missed: high albumin (low is late and unusual), high lipids, low alkaline phosphatase, low urea and creatinine. Patients who over-exercise and are bradycardic may also be dismissed as having healthy athletes’ hearts when the bradycardia is a result of malnutrition and does not respond to exertion as athletes’ hearts do. Therefore, joint working and joint decision-making is particularly important. Furthermore, it may be appropriate to prescribe psychiatric medications, to help reduce the patient's anxiety and distress; it would be advisable for paediatricians to do this in consultation with psychiatrists, bearing in mind the increased likelihood of side-effects.

### Multidisciplinary working

In this case, if the patient became medically unstable to the extent that the paediatric team felt her life was at risk, then the team would have to consider NGT feeding against her will as a lifesaving intervention, under the appropriate legal framework. This decision should not be taken lightly and would require agreement from both the paediatric team and CAMHS team in discussion with the patient's parents, as well as mobilisation of the appropriate staff to support the safe delivery of hydration and nutrition under restraint.

If there was a life-threatening deterioration in this young person's physical health while awaiting the MHA assessment, paediatricians must act ‘in the best interest of the child’ as set out in the Children Act 2004.^19^ This means that NGT feeding under physical restraint, against the young person's wishes, can be done to save her life.

### Considering different types of restraint

There is a broad spectrum of physical restraint, ranging from holding the patient's hands for reassurance, to several staff securing the head, arms and legs. Physically restraining patients is usually beyond the safe practice of paediatric nursing teams, and therefore the staffing mix would have to be considered, to include restraint-trained mental health nurses in sufficient numbers, to safely deliver this intervention.

## Conclusion

To date, there is little research regarding NGT feeding under physical restraint. However, this scenario outlines a situation where the practice would potentially be appropriate, when a young person's life is at risk. This paper has outlined the legal, ethical and practical considerations that clinicians should be aware of.

## About the authors

**Sarah J. Fuller** is an Advanced Specialist Eating Disorders Dietitian with East London NHS Foundation Trust, UK; and a Research Dietitian at the Child and Adolescent Mental Health Department, Imperial College London, UK. **Simon Chapman** is a Consultant in Paediatric and Adolescent Medicine at the Department of Paediatrics, King’s College London, UK. **Emma Cave** is a Professor of Healthcare Law at the Durham Law School, Durham University, UK. **James Druce-Perkins** is a Highly Specialist Dietitian with the Child and Adolescent Mental Health Department, Aneurin Bevan University Health Board, UK. **Poppy Daniels** is an expert by experience. **Jacinta Tan** is a Consultant Child and Adolescent Psychiatrist with Oxford Health NHS Foundation Trust, UK; and Senior Clinical Research Fellow at the Department of Psychiatry, Oxford University, UK.
